# Artificial Intelligence-Driven Call Center Operations in a High-Volume Neurology Practice: Impact on Access, Efficiency, Revenue Growth, and Cost Containment

**DOI:** 10.7759/cureus.112227

**Published:** 2026-07-07

**Authors:** Patricio S Espinosa, Yaira R Espinosa, Juan P Espinosa

**Affiliations:** 1 Neurology, Espinosa Neuroscience Institute, Boca Raton Regional Hospital, Boca Raton, USA; 2 School of Business, University of Miami, Boca Raton, USA; 3 Health Sciences, Espinosa Neuroscience Institute, Boca Raton, USA

**Keywords:** artificial intelligence in healthcare, call center automation, healthcare administration, healthcare operations, neurology practice management

## Abstract

Background

Healthcare systems face increasing administrative burdens, workforce shortages, rising labor costs, and growing patient expectations for rapid communication. Telephone call management remains one of the most labor-intensive nonclinical functions in outpatient medicine. Artificial intelligence (AI)-driven voice assistants have emerged as a potential solution to improve patient access while reducing operational costs. Beyond cost savings, AI communication platforms may increase revenue by improving the capture of new patient consultations and diagnostic referrals otherwise lost to communication delays.

Objective

To evaluate the operational, financial, and revenue impact of implementing an AI-powered telephone answering and patient communication system in a high-volume outpatient neurology practice, emphasizing patient access, referral capture, and new patient acquisition.

Methods

A retrospective, observational pre-post cohort study was conducted at Espinosa Neuroscience Institute, a practice of three neurologists and four advanced practice providers. After deploying an AI-powered call center platform, inbound communications - including appointment scheduling, demographic intake, insurance collection, FAQs, message routing, and patient triage - were managed through a Health Insurance Portability and Accountability Act (HIPAA)-compliant automated voice interface. The study evaluated operational metrics, staffing requirements, call volume, communication backlog, referral capture, and estimated labor costs by comparing a 90-day pre-implementation period (December 2025-February 2026) to an 86-day post-implementation period (March-May 2026). As a retrospective analysis of de-identified data for quality improvement, it was exempt from Institutional Review Board (IRB) approval.

Results

The practice receives 400-500 inbound calls daily. Before AI implementation, unresolved communication queues frequently exceeded 500 messages, with patients reporting prolonged wait times and delayed responses. Following deployment, all incoming calls could be answered simultaneously without queue limitations. During the first 86 days, the AI platform processed communications representing 5,484 unique patient encounters and managed an average of 63.8 new patient interactions per day. Communication backlogs were reduced by over 98%, with fewer than 10 active conversations open at any given time. Eliminating communication bottlenecks improved the capture of new patient consultations and referrals for neurological evaluations, EEG, EMG, and MRI. Requests previously lost to unanswered calls or excessive hold times were more consistently scheduled and completed, resulting in increased utilization of clinical and diagnostic services. Financial modeling estimated annual labor cost savings of approximately $216,500 while creating new revenue opportunities through improved referral conversion and patient acquisition.

Conclusions

Implementing an AI-powered call center significantly improved patient access, reduced communication backlogs, increased operational efficiency, and lowered staffing costs in a high-volume neurology practice. Beyond cost containment, the system enhanced new patient acquisition and referral capture, generating new lines of business through increased utilization of neurological and diagnostic services. AI-assisted communication represents a scalable strategy for improving financial sustainability and patient access in outpatient healthcare.

## Introduction

Healthcare organizations face unprecedented administrative challenges. Rising labor costs, workforce shortages, increasing patient volumes, and growing documentation requirements have created significant operational burdens for outpatient practices. Among these challenges, telephone communication remains one of the most resource-intensive functions and one of the most consequential for both patient safety and practice revenue [1].

Traditional call centers rely on human personnel to answer, route, and document patient communications. While effective, these systems are inherently limited by staffing availability. A single employee can process only one call at a time, resulting in delays, abandoned calls, and patient dissatisfaction during periods of high demand. In a neurology practice, the consequences of missed calls extend well beyond inconvenience and financial loss; they carry direct implications for patient safety and clinical quality [2].

The scope of the problem: what missed calls cost a neurology practice

Prior to AI implementation, the Espinosa Neuroscience Institute faced a broad and compounding set of communication failures that affected every dimension of practice operations. These problems can be organized into five distinct categories, each carrying its own clinical and financial consequences.

Urgent Patient Calls

Patients experiencing acute neurological symptoms - including new-onset seizures, sudden severe headache, acute weakness, altered mental status, or medication side effects - frequently attempted to reach the practice by telephone during business hours. When call volumes exceeded staff capacity, these urgent calls were placed in queue alongside routine scheduling requests, often resulting in prolonged hold times or call abandonment. Patients who could not reach a provider in a timely manner were left without guidance, potentially delaying necessary emergency evaluation or resulting in unnecessary emergency department visits. The inability to reliably triage urgent calls in real time represented a significant gap in the continuum of neurological care.

Communications From External Healthcare Partners

The practice routinely receives inbound calls from radiology centers, clinical laboratories, pharmacy benefit managers, and other healthcare facilities. These calls frequently carry time-sensitive clinical information, including reports of abnormal imaging findings, critical laboratory values, and urgent consultation requests from hospital teams. When these calls went unanswered or were routed to voicemail during peak hours, the resulting communication delays had the potential to affect clinical decision-making and patient outcomes. Radiology centers reporting incidental findings on brain MRI or spine imaging, for example, require prompt acknowledgment and follow-up coordination; delays in processing these communications risk clinical harm and medicolegal exposure.

Prescription Refill Requests

Neurological patients frequently require ongoing management of chronic medications, including antiepileptic drugs, migraine prophylaxis agents, medications for movement disorders, and other long-term neurological therapies. Prescription refill requests - whether originating directly from patients or routed through pharmacy systems - require timely processing to prevent treatment interruptions. Under the prior staffing model, high call volumes resulted in refill requests being delayed, misrouted, or lost entirely within communication queues. Patients who ran out of critical medications due to processing delays faced the risk of seizure recurrence, rebound headache, or exacerbation of their underlying neurological condition.

Insurance Prior Authorization Requirements

Insurance prior authorization requirements for neurological medications, diagnostic studies, and procedures represent one of the most administratively burdensome aspects of specialty practice management. Pharmacy benefit managers and insurance carriers initiate prior authorization workflows via telephone and fax, often requiring timely responses to avoid delays in patient care. Under the previous communication model, prior authorization calls from insurance companies and pharmacy benefit managers were frequently missed or inadequately documented, resulting in delayed approvals, treatment interruptions, and additional administrative rework. The cumulative burden of these failures contributed to staff burnout and reduced the time available for direct patient care activities [3].

New Patient Consultations and Referral Capture

New patient consultations, referrals for neurological evaluations, and requests for diagnostic studies, including electroencephalography (EEG), electromyography (EMG), and magnetic resonance imaging (MRI), were frequently delayed or lost entirely because of communication bottlenecks. The revenue impact of these missed opportunities, while difficult to quantify precisely, represented a high and largely invisible cost to the practice. Research has demonstrated that call center performance - specifically average speed of answer and call abandonment rates - is directly associated with patient perceptions of access and satisfaction. Improving telephone responsiveness, therefore, carries both clinical and financial implications [4].

Collectively, these five categories of communication failure illustrate that the consequences of inadequate call center capacity in a specialty neurology practice are not merely operational - they are clinical, financial, and reputational. The need for a scalable, reliable, and intelligent communication solution was therefore both urgent and multidimensional.

Recent advances in conversational artificial intelligence have enabled healthcare organizations to deploy voice assistants capable of answering multiple simultaneous calls, collecting structured patient information, providing standardized responses, scheduling appointments, and routing requests appropriately. The Espinosa Neuroscience Institute implemented an AI-powered communication platform to address these compounding communication challenges. This report describes the operational, financial, and clinical quality outcomes associated with this transition, incorporating real-world data from the first three months of system deployment [5,6].

## Materials and methods

Study design and ethics approval

A retrospective, observational pre-post cohort study was conducted to evaluate the operational impact of an AI-powered communication platform. Operational metrics from a 90-day pre-implementation period (December 2025 through February 2026) were compared with those from an 86-day post-implementation period (March 2026 through May 2026) following deployment of the AI platform. These nearly equivalent observation periods were intentionally selected to provide comparable baseline and post-intervention assessments while minimizing the effects of seasonal variation in call volume and practice operations. As this study involved retrospective analysis of de-identified operational data collected for quality improvement and practice management purposes, it was exempt from Institutional Review Board (IRB) approval.

Setting

The study was conducted at a private outpatient neuroscience practice (Espinosa Neuroscience Institute) consisting of three board-certified neurologists, four advanced practice providers, and dedicated clinical and administrative support staff totaling more than 30 active system users.

Technical architecture and workflow

During the study period, the AI-powered communication platform (Freed Front Desk; Freed AI, Inc., San Francisco, CA, USA) was implemented using carrier forwarding from the practice's primary telephone line. All inbound calls were initially answered by the AI platform, which collected demographic and insurance information, identified the reason for the call, answered routine administrative questions using a customized knowledge base, and routed communications to the appropriate departmental inbox. At the time of this study, the platform was not directly integrated with the electronic health record (EHR) or practice management system for autonomous scheduling or documentation. Appointment scheduling, EHR documentation, prescription processing, and other clinical or administrative tasks requiring access to the medical record were completed by clinic staff after AI-assisted intake and routing. Consequently, the operational efficiencies reported in this study reflect automation of communication management and workflow optimization rather than fully autonomous EHR-integrated practice operations.

AI platform deployment

The AI-powered communication platform (Freed Front Desk) was deployed through AT&T carrier forwarding to a dedicated telephone line and operated continuously 24 hours per day, seven days per week, 365 days per year throughout the study period. Unlike traditional human-staffed call centers, the system remained continuously available without interruptions related to staffing schedules, breaks, shift changes, weekends, holidays, or after-hours coverage limitations. As a result, all inbound calls could be answered at any time of day or night, regardless of clinic operating hours.

The system was programmed to answer multiple inbound calls simultaneously using a standardized conversational workflow, collect complete patient demographics (full name, date of birth, callback number, email address, and mailing address), capture insurance information and reason for contact, schedule appointments across all providers, and route clinical and administrative messages to the appropriate departmental inbox (Front Desk, Clinical Team, or Billing Department).

The platform also provided office directions, hours of operation, physician specialty information, and practice-specific guidance through a customized 21-entry knowledge base. Topics included accepted insurance plans, Medicaid eligibility, fax numbers, provider locations, on-call protocols, medication prior authorization procedures, hospital consultation workflows, and available service lines, including infusion therapy, medical weight management, concierge neurology services, and neurological diagnostic testing.

Call management workflow

During the study period, all inbound telephone calls were initially answered by the AI-powered communication platform, which functioned as the primary point of contact for the practice. The AI collected demographic and insurance information, determined the purpose of the call, and attempted to complete the interaction autonomously whenever possible. Routine administrative requests, including appointment scheduling, office information, provider information, demographic collection, and message routing, were managed directly by the AI. Calls requiring clinical judgment, urgent medical triage, complex scheduling conflicts, prescription issues requiring provider review, insurance appeals, or other circumstances beyond the AI's programmed capabilities were escalated to the appropriate clinic staff member (front desk, clinical team, or billing department) for human intervention. Consequently, all 400-500 daily inbound calls were initially processed by the AI platform, with only a subset requiring transfer to clinic personnel for completion.

The AI platform was configured to function as an administrative communication assistant rather than an autonomous clinical decision-maker. Calls were screened using predefined conversational workflows to identify potentially urgent neurological symptoms or requests requiring clinical judgment. Communications involving suspected neurological emergencies, medication-related concerns requiring provider input, or other clinically complex issues were immediately escalated to the appropriate clinical staff. Callers also had the ability to request transfer to a human representative at any point during the interaction, and these requests were honored without restriction. The AI did not provide independent medical diagnoses or treatment recommendations.

Data source and metric definitions

Operational data were obtained directly from the Freed Front Desk analytics dashboard for the 86-day post-implementation period and were compared with routine practice management reports and communication metrics from the 90-day period preceding implementation. The data extraction process involved exporting aggregated system logs and call transcripts, which were validated against the EHR scheduling system to ensure accuracy.

Key metrics evaluated in this study were defined as follows: Backlog (unresolved message queue): The total number of inbound communications (calls, voicemails, messages) that had been received but not yet addressed, routed, or resolved by staff at the end of a business day; Same-day resolution: The percentage of inbound patient inquiries or requests that were fully addressed and closed within the same business day they were received; Active conversations: The number of ongoing, unresolved communication threads requiring staff intervention at any given point in time; Unique communication encounters: The 5,484 encounters reported during the post-implementation period represent unique communication episodes (telephone calls or message threads) processed by the AI platform. These encounters do not necessarily represent unique individual patients, as the same patient may have contacted the practice multiple times during the study period. The dataset includes both new patient appointment requests and communications from established patients, caregivers, referring providers, pharmacies, and other healthcare partners.

Methodology for the 837-call analysis

To evaluate the distribution of call types and estimate the potential for automation, a sample of 837 inbound calls was selected from the AI platform's archived conversations recorded during May and June 2026. Calls were selected using a stratified sampling approach designed to provide representative coverage across weekdays, weekends, business hours, and after-hours periods. Within each stratum, calls were reviewed without regard to call outcome or complexity. Two independent administrative reviewers analyzed the call transcripts and recordings and categorized each interaction into predefined call types (e.g., appointment management, prescription requests, medical records, insurance inquiries, urgent clinical calls). Disagreements were resolved through consensus with a third senior reviewer. Automation potential was estimated following manual review of each call by two independent reviewers and reflects the proportion of calls that, based on the AI platform's demonstrated capabilities and anticipated functionality with full integration into the EHR, scheduling, pharmacy, and billing systems, could reasonably be managed without human intervention. These estimates represent expert consensus projections rather than prospectively validated performance metrics.

## Results

Operational improvements

Prior to AI implementation, the practice managed 400-500 inbound calls per day with a predominantly human-staffed front desk. Communication backlogs were a persistent problem, with unresolved message queues frequently exceeding 500 items and patients reporting wait times that discouraged follow-through on appointment requests. The operational and clinical consequences of this bottleneck included delayed care, reduced patient satisfaction, and - critically - lost new patient and referral revenue (Table 1).

**Table 1:** The operational state before and after AI deployment Data demonstrate stable call volume with substantial improvements in communication efficiency, including increased call-handling capacity, reduced message backlog, improved same-day resolution, and enhanced patient engagement during the 86-day post-implementation period.

Metric	Pre-implementation	Post-implementation (86 Days)
Daily Inbound Call Volume	400-500	400-500
Simultaneous Call Capacity	1 per agent	Unlimited
Peak Unresolved Message Queue	>500	<10
Same-Day Resolution Rate	Low (<50%)	High (>90%)
Unique Communication Encounters (total)	Unknown	5,484
Average Communication Encounters per Day	Unknown	~63.8
Active Open Conversations	>500	<10

Following the deployment of the AI platform in March 2026, the practice transitioned to simultaneous, unlimited call-answering capacity. Within the first 86 days of operation, the AI platform processed 5,484 communication encounters, averaging 63.8 encounters per day. These encounters included new patient appointment requests, established patient communications, referral-related calls, and other administrative or clinical interactions. Although the system substantially improved the capture of new patient consultations, the reported encounter count should not be interpreted as the number of newly registered patients. A new patient inquiry was defined as a communication initiated by an individual who was not previously registered in the practice and who contacted the clinic to request an initial neurological consultation or diagnostic service (e.g., EEG, EMG, or MRI). The AI platform identified these requests during the intake process; however, this study did not link communication records to EHR scheduling data to determine whether each inquiry resulted in a completed clinic visit. The current open communication backlog across all inboxes averages fewer than 10 active conversations at any given time. These remaining conversations do not represent unanswered calls or communication failures; rather, they reflect interactions that appropriately remain open while awaiting physician review, patient response, insurance authorization, coordination with external healthcare providers, or other administrative or clinical actions requiring human follow-up. Thus, the remaining active conversations represent ongoing workflow rather than unresolved communication backlog.

New patient acquisition and referral capture

One of the most clinically and financially significant outcomes of AI implementation has been the marked improvement in new patient acquisition and referral capture. Prior to deployment, a substantial proportion of new patient calls - particularly those arriving during peak hours or after brief hold periods - were abandoned before reaching a staff member. Research has shown that at least 60% of patients will abandon a call if placed on hold for longer than one minute, and that telephone access barriers directly influence whether patients seek care at a given practice. Referring physicians and their staff, accustomed to immediate response, frequently redirected referrals when calls went unanswered.

Following AI deployment, every inbound call is answered immediately and without exception. New consultation requests, referrals for neurological evaluations, and requests for diagnostic studies - including EEG, EMG, nerve conduction studies, and MRI - are now captured, documented, and routed for scheduling in real time. This improvement in referral capture has opened new lines of business for the practice, expanding both the volume and diversity of clinical services delivered. Prior studies have demonstrated that automated patient navigation platforms can increase referral conversion rates and scheduling efficiency significantly, with one study reporting a 30% improvement in referral-to-appointment conversion within two business days following automated platform deployment [4].

The AI assistant currently performs the following functions (see Table 2).

**Table 2:** Current functions of the AI assistant Summary of administrative, scheduling, information management, and patient communication functions performed by the AI platform during the study period. The system was designed to automate routine front desk operations, facilitate patient access, and support communication workflows within a high-volume outpatient neurology practice. DOB: date of birth; FAQ: frequently asked questions; HIPAA: Health Insurance Portability and Accountability Act

Function	Capability
Appointment Scheduling	Yes
Demographic Collection	Yes
DOB Verification	Yes
Insurance Collection	Yes
Office Information	Yes
Physician Information	Yes
Specialty Information	Yes
Message Routing	Yes (Front Desk, Clinical, Billing)
FAQ Responses	Yes (21+ Knowledge Base Entries)
Simultaneous Call Handling	Unlimited
HIPAA-Compliant Documentation	Yes
New Patient Intake	Yes
Referral Capture and Routing	Yes
Diagnostic Study Scheduling (EEG, EMG, MRI)	Yes
Urgent Call Triage and Escalation	Yes
Prescription Refill Request Routing	Yes
Prior Authorization Call Routing	Yes
External Partner Communication Routing (Radiology, Lab, Pharmacy)	Yes

The AI system's ability to identify the specific nature of each call - categorizing requests as Scheduling, Referral, Urgent, Billing, Medical Records, Pharmacy, or Escalation - ensures that high-value referral calls are immediately flagged and processed by the appropriate clinical or administrative team member, minimizing the risk of revenue leakage.

A comprehensive analysis of 837 randomly selected inbound calls collected during May and June 2026 reveals the distribution of call types and their respective automation potential. The sample data were randomly selected from conversations occurring during this two-month period, ensuring representative coverage of seasonal and temporal variations in call patterns. The analysis demonstrates that appointment management dominates call volume at 45.2%, followed by prescription/pharmacy requests at 15.5% and administrative information requests at 18.0%. Notably, 18% of all calls (150 calls) are simple information requests requiring no clinical judgment and can be fully automated with 95-100% success rates, representing the highest immediate opportunity for operational efficiency improvement. When combined with hybrid automation opportunities in scheduling, prescription management, and medical records processing, the data indicate that 50-65% of all inbound calls can be effectively managed by AI systems with appropriate integration to practice management, pharmacy, and billing systems, while 35-50% of calls require human intervention due to clinical complexity or escalation requirements. The reported automation potential percentages should be interpreted as expert estimates of the proportion of calls that could be managed autonomously under the described implementation and are not direct measurements of observed AI performance (Table 3).

**Table 3:** Inbound call classification and automation potential Distribution of inbound calls by category, including appointment management, prescription requests, medical records, office information, provider information, insurance inquiries, contact information, fax requests, urgent clinical calls, and other requests. The table also summarizes the percentage of total call volume represented by each category and the estimated potential for automation versus human intervention. Automation potential was estimated by expert review and reflects projected capability under full system integration; it does not represent prospectively measured automation success rates.

Call Category	Count	%	Automation Potential	Primary Drivers	Human Intervention Required
Appointment Management	378	45.2%	60-70%	New scheduling, rescheduling, cancellations	Complex conflicts
Prescription/Refill	130	15.5%	50-60%	Medication refills, pharmacy coordination, insurance	Denials, urgent needs
Medical Records	55	6.6%	40-50%	Third-party requests, records transfers, documentation	Complex authorizations
Office Location and Directions	50	6.0%	95-100%	Address inquiries, directions, navigation	None - fully automatable
Provider Information	42	5.0%	70-80%	Provider requests, availability, referrals	Clinical recommendations
Insurance Inquiry	37	4.4%	40-50%	Prior authorization, coverage verification	Complex appeals
Contact Information	32	3.8%	90-95%	Callback requests, message relay, contact details	None - fully automatable
Fax Number Requests	31	3.7%	95-100%	Fax requests, transmission coordination	None - fully automatable
Clinical/Urgent	12	1.4%	10% (triage only)	Emergency situations, hospital coordination	All urgent handling
Other/Unclear	70	8.4%	30-40%	Incomplete requests, ambiguous needs, wrong numbers	Clarification required
Total	837	100%	50-65%	-	35-50%

Financial analysis

Estimated Staffing Requirements Without AI

Based on current communication volume, the practice estimates a need for approximately eight full-time call center personnel to manage the equivalent inbound call volume without AI support. Administrative costs in US healthcare are substantial, with estimates suggesting that administrative spending reached approximately $950 billion in 2019, representing 15-25% of total national health expenditures (see Table 4) [1].

**Table 4:** Estimated annual personnel salary costs (direct salary expenses) Staffing and financial impact of AI implementation. Comparison of estimated staffing requirements and labor costs before and after deployment of the AI-powered communication platform. The table summarizes projected personnel expenses under a traditional human-staffed model versus an AI-assisted workflow, including platform costs, estimated annual labor savings, additional reductions in administrative burden, and total projected annual cost savings. FTE: full-time equivalent

Cost Category	Estimated Annual Cost (USD)
Pre-AI model (8 FTE at $35,000-40,000/FTE annually)	~$280,000-320,000
Post-AI model (3 FTE + AI platform: $105,000-120,000 + $24,000)	~$129,000-144,000
Estimated annual labor savings	$151,000-191,000
Additional savings from reduced administrative burden	~$25,000-50,000
Total estimated annual savings	~$176,000-241,000

The short-term and long-term financial impact of AI implementation, including estimated annual savings and five-year cost projections, is summarized in Table 5.

**Table 5:** Five-year financial projection using fully loaded personnel costs (salary, benefits, payroll taxes, and annual wage growth)

Year	Traditional Staffing Model	AI-Assisted Model	Annual Savings
1	$384,800	$168,300	$216,500
2	$396,344	$168,300	$228,044
3	$408,234	$168,300	$239,934
4	$420,481	$168,300	$252,181
5	$433,095	$168,300	$264,795

Financial Summary: Total Five-Year Labor Savings: Approximately $1.2 Million

Table 4 summarizes direct annual salary expenditures, whereas Table 5 models total personnel costs over five years, incorporating employee benefits, payroll taxes, and projected annual salary inflation. Therefore, the baseline costs differ between the two tables.

Revenue impact of new patient and referral growth

In addition to direct labor cost savings, the practice has experienced a meaningful increase in revenue attributable to improved new patient capture and referral processing. Prior to AI implementation, missed calls and communication delays resulted in lost consultations and diagnostic studies. Following deployment, the consistent capture of new patient requests and referrals for EEG, EMG, MRI, and neurological consultations has expanded the practice's active patient base and diagnostic service volume.

While precise pre- and post-implementation revenue comparisons require further longitudinal analysis, the revenue contribution of each captured new patient consultation - combined with associated downstream diagnostic testing - represents a substantial multiplier effect on the financial return of AI investment. The $2,000 monthly platform cost is therefore offset not only by labor savings but also by incremental revenue generated through previously unrealized clinical activity.

## Discussion

This case study demonstrates that AI-powered communication systems can substantially improve operational performance, patient safety, and clinical quality in outpatient healthcare environments. Real-world data from the first 86 days of deployment at Espinosa Neuroscience Institute confirms that the system effectively manages a high volume of patients - over 5,400 encounters in fewer than three months - while nearly eliminating communication backlogs. These findings are consistent with the broader literature demonstrating that conversational AI platforms can facilitate patient interviews, support documentation, and enhance real-time clinical dialogue at scale [2].

Of particular clinical significance is the system's ability to address the full spectrum of communication failures that characterized the pre-implementation environment. Urgent patient calls are now answered immediately and triaged in real time, with escalation protocols ensuring that clinically time-sensitive requests are routed directly to the appropriate provider. Communications from radiology centers, clinical laboratories, and pharmacy benefit managers - previously subject to delays during peak call hours - are now captured, documented, and routed without exception. Prescription refill requests are processed through a structured intake workflow, reducing the risk of medication interruptions for patients on critical neurological therapies. Prior authorization calls from insurance carriers are logged and directed to the billing and clinical teams for timely response, reducing administrative rework and treatment delays. Each of these improvements represents not only an operational gain but a meaningful enhancement in the quality and safety of patient care.

Patient safety remains paramount when implementing AI-assisted communication systems. In our practice, the AI platform was intentionally designed to support administrative efficiency while maintaining clinician oversight for situations requiring medical judgment. By immediately answering all inbound calls and rapidly identifying potentially urgent communications for escalation, the system may reduce delays associated with communication bottlenecks. Although no AI-related adverse events or missed urgent neurological presentations were identified during the study period, larger prospective studies with formal safety outcome measures are needed to determine the impact of AI-assisted communication on patient morbidity, emergency department utilization, and other clinical outcomes.

Unlike traditional call centers, AI systems are not constrained by the one-call-per-employee limitation. Multiple patient interactions can be processed simultaneously, reducing wait times and improving accessibility. The system's ability to accurately categorize calls and route them to specific departments ensures that human staff handle only tasks requiring clinical judgment or complex intervention, while routine scheduling, intake, and informational requests are resolved autonomously. This redistribution of administrative labor is particularly valuable in the context of the ongoing healthcare workforce shortage, with projections estimating a significant deficit of healthcare workers in the coming years [7,8]. The AI platform was designed to augment, rather than replace, clinic personnel by autonomously managing routine administrative communications while seamlessly escalating interactions requiring human expertise or clinical decision-making.

The revenue implications of this transformation deserve particular emphasis. In specialty neurology practice, new patient consultations and diagnostic referrals represent high-value encounters. Neurological conditions are the leading cause of combined disability and mortality globally, creating sustained and growing demand for neurological services. When communication failures prevent these encounters from being scheduled, the financial loss is real but often invisible to practice leadership. AI-powered call management makes this loss recoverable by ensuring that every referral call is answered, every new patient request is captured, and every diagnostic study request is routed for timely scheduling.

The detailed call classification analysis reveals that administrative and information requests represent the highest automation opportunity, with 18% of calls being fully automatable with 95-100% success rates. This finding suggests that further optimization through system integration with practice management, pharmacy, and billing platforms could increase overall automation rates from the current 40-50% to 50-65%, generating additional annual savings of $40,000-80,000 while further improving patient access and staff efficiency.

The practice has effectively opened new lines of business - not by adding services, but by ensuring that existing service lines are accessible to every patient who attempts to reach the practice. Improved responsiveness has also enhanced the practice's reputation among referring physicians, reinforcing referral relationships that drive long-term revenue growth. Prior research has confirmed that improvements in telephone triage and communication systems lead to measurable gains in patient satisfaction and appointment accessibility [5,6].

An important advantage of AI-assisted communication is its ability to scale with increasing call volume without creating additional telephone queues. Although the platform cannot expand the clinical capacity of healthcare providers, it ensures that every incoming communication is answered, prioritized, and routed appropriately. During periods of high demand, the AI maintains communication quality by resolving routine administrative requests, collecting complete patient information, identifying urgent cases for immediate escalation, and facilitating scheduling, waitlist management, or referral to alternative resources when provider capacity is exceeded. Thus, the system addresses communication bottlenecks while preserving appropriate clinical oversight and patient access.

One of the most clinically and financially significant outcomes of AI implementation has been the marked improvement in new patient acquisition and referral capture. Prior to deployment, a substantial proportion of new patient calls - particularly those arriving during peak hours or after prolonged hold times - were abandoned before reaching a staff member. Previous studies have demonstrated that telephone access barriers, including long wait times and high call abandonment rates, are associated with reduced patient satisfaction, decreased appointment scheduling, and loss of referral opportunities. Improving telephone responsiveness has therefore been recognized as an important determinant of patient access and practice performance [5].

As healthcare labor costs continue to rise and patient expectations for immediate access intensify, AI-assisted communication systems may become an increasingly indispensable component of sustainable and competitive outpatient practice management [9]. Future integration of conversational AI with EHR and practice management systems may permit fully automated scheduling, documentation, and administrative workflows, further increasing efficiency beyond that observed in the present study.

Limitations

This analysis is based on operational data from a single neurology practice over an initial three-month deployment period, with supplementary call classification data collected during May and June 2026. The retrospective nature of the study and the reliance on system-generated analytics limit the ability to draw definitive causal conclusions. Formal quantification of revenue attributable to new patient capture, patient satisfaction surveys, detailed call duration analysis, and multicenter validation studies remains necessary. Additional research should evaluate quality metrics, patient satisfaction, referral source analysis, and long-term operational and financial outcomes.

Although the AI platform captured new patient inquiries and referral requests, this study did not link communication records to EHR scheduling data. Consequently, the proportion of inquiries that resulted in completed clinic visits, completed diagnostic studies, or downstream revenue could not be determined. Future studies incorporating EHR integration will evaluate referral conversion rates, appointment completion, patient retention, and associated financial outcomes.

## Conclusions

Implementation of an AI-powered call center in a high-volume neurology practice resulted in significant reductions in communication backlog, improved same-day patient responsiveness, enhanced access to care, and estimated annual labor cost savings exceeding $200,000. Beyond cost containment, the system resolved a broad set of pre-existing communication failures - including missed urgent patient calls, delayed communications from radiology centers and clinical laboratories, lost prescription refill requests, unanswered prior authorization calls, and unreliable routing of external healthcare partner communications - all of which had previously posed risks to patient safety and clinical quality. The system also enabled a measurable increase in new patient acquisition and referral capture-including consultations, EEG, EMG, MRI, and neurological evaluations - generating new lines of business that contribute directly to practice revenue.

Operational data from the first 90 days confirm the system's ability to seamlessly manage thousands of patient interactions, demonstrating that AI-assisted communication delivers value not only through expense reduction but also through revenue generation, improved patient safety, and enhanced quality of neurological care. Detailed analysis of call types reveals significant opportunities for further optimization through system integration, with potential to increase automation rates to 50-65% and generate additional annual savings of $40,000-80,000. These findings support the growing role of artificial intelligence as a transformative administrative tool capable of improving healthcare delivery, strengthening practice finances, and expanding access to neurological care.
